# Building safe surgery knowledge and capacity in Cambodia: a mixed-methods evaluation of an innovative training and mentorship intervention

**DOI:** 10.1080/16549716.2021.1998996

**Published:** 2021-12-20

**Authors:** Sehrish Bari, Joseph Incorvia, Olivia Ahearn, Lem Dara, Swati Sharma, John Varallo, Victoria Smith, Monica Cainer, Cheav Samphy, Kith Rathamony, Ngin Kanora, Vithiea Dara, John G Meara, Virya Koy, Shehnaz Alidina

**Affiliations:** a Program in Global Surgery and Social Change, Harvard Medical School, Boston, MA, USA; b Calmette Hospital, Phnom Penh, Cambodia; c Dalberg Global Development Advisors Singapore; d Safe Surgery, Jhpiego, Baltimore, Maryland, USA; e Assist International, Ripon California, California; f Department of Plastic and Oral Surgery, Boston Children’s Hospital, Boston, MA, USA; g Department of Hospital Services, Ministry of Health, Phnom Penh, Cambodia

**Keywords:** Global surgery, monitoring and evaluation in safe surgery, surgery in Cambodia, surgical safety checklist, surgical capacity building

## Abstract

**Background:**

Working in partnership with the Cambodian Ministry of Health, the Safe Surgery 2020 initiative (SS2020) supports the prioritization of surgery and mobilization of resources to target limited workforce capacity. An evaluation study was conducted to assess the impact of SS2020 on intervention hospitals in Cambodia.

**Objective:**

To understand the impact of the SS2020 program on intervention hospitals in Cambodia by assessing the changes in key surgical performance indicators before and after the intervention, identifying key barriers and facilitators to adoption of learnings, and discovering lessons on the uptake and diffusion of this initiative in Cambodia and other similar contexts.

**Methods:**

This study is a convergent mixed-methods evaluation of a one-year multicomponent SS2020 intervention. Surgical observations were conducted in 8 intervention hospitals at baseline and endline to evaluate pre and post adherence to 20 safety, teamwork, and communication items. Fifteen focus groups were conducted in all intervention sites at endline to assess key facilitators and barriers to positive impact.

**Results:**

There was significant improvement in 19 of 20 indicators assessed during surgical observations. Among the highest performing indicators were safety items; among the lowest were communication items. Participants self-reported improved knowledge and positive behavior change after the intervention. Institutional change and direct patient impact were not widely reported. Most participants had favorable views of the mentorship model and were eager for the program to continue implementation.

**Conclusions:**

The results provide evidence that change in surgical ecosystems can be achieved on a short timeline with limited resources. The hub-and-spoke mentorship model can be successful in improving knowledge and changing behavior in surgical safety. Workforce development is important to improving surgical systems, but greater financial and human resources are needed. Ministry support in adopting, leading, and scaling is crucial to the continued success of safe surgery interventions in Cambodia.

## Background

In 2015, the Lancet Commission on Global Surgery reported that only 6% of the 313 million surgical procedures performed annually are within the poorest one-third of the world [1]. High case-fatality rates from treatable surgical conditions are largely attributed to the unmet surgical need in these regions. Southeast Asia is one of the regions with the greatest unmet need, with a reported 2,045 per 100,000 population or 12.5 million additional surgical procedures required each year [1]. Moreover, an estimated 81% of individuals in Southeast Asia lack access to safe, affordable, surgical, obstetric, and anesthesia care, in comparison to 3.6% in higher-income countries [2]. Disease Control Priorities reported that approximately 294,730 (2.3%) of deaths could be prevented per year in East Asia and Pacific if basic surgical care was provided [3].

Cambodia is a southeast Asian nation with a population of 16.2 million and is bordered by Thailand, Laos, and Vietnam as well as the Gulf of Thailand [4,5]. After more than two decades of economic growth, Cambodia became a lower middle-income country in 2015. However, key reforms are still needed in both health and education [6]. The 2015 Health System Review reported a physician workforce of 1.51 per 10,000 population (2,157) and a physician specialist workforce of 0.18 per 10,000 population (259) [7]. Furthermore, 74.4% of specialist and 40.3% of generalist medical practitioners were concentrated at the central level, including six national hospitals in Phnom Penh [7].

Limited data exists on the current state of Cambodia’s surgical system. Although the Health Development Goals and Targets of Cambodia’s 2016–2020 Health Strategic Plan (HSP) included goals to improve maternal and child health, communicable diseases, non-communicable diseases, and accidents and injuries, there was no mention of surgery [8]. The only surgical indicators selected for monitoring and evaluation of HSP progress were abortion rate, cesarean section rate (% of live births), and cataract surgical rate per 1,000,000 population [8]. The World Bank reported a surgical workforce in Cambodia of 4 per 100,000 population, far from the Lancet recommendation of 20 surgical, obstetric, and anesthetic physicians per 100,000 population [1,9]. With an estimated 419 surgical procedures performed per 100,000 population, Cambodia’s surgical capacity is well below the Lancet Commission’s recommendation of a minimum operative value of 5000 surgical procedures per 100,000 population [1,10]. An evident gap in Cambodia’s HSP is the lack of measurement around surgical quality and safety. There is limited available data that provides insights into surgical quality in Cambodia; this paper is one of the first to address this gap.

Funded by the GE Foundation, Safe Surgery 2020 (SS2020) is a global initiative comprised of a multi-stakeholder partnership aimed at improving surgical and health-care systems in developing countries [10]. Working in partnership with the Cambodian Ministry of Health (MoH), SS2020 supports the prioritization of surgery and mobilization of resources to target limited workforce capacity. The program uses a multicomponent intervention focusing on capacity building and mentorship, whereby surgical teams are empowered through leadership development and trained on specific clinical skills, such as safe anesthesia and obstetric care, with ongoing mentorship. This curriculum was modeled after previously implemented Safe Surgery 2020 programs in Tanzania and Ethiopia [10].

The aim of this study was to conduct a mixed-methods evaluation of SS2020 interventions implemented in hospitals in 2019 to understand the overall impact of the SS2020 program. This study was designed to answer three key research questions: what are the changes in key surgical performance indicators before and after the SS2020 intervention; what were key barriers and facilitators to adoption of intervention learnings, and what lessons can we extract for the uptake and diffusion of this safe surgery initiative in Cambodia and other similar contexts? Results will inform future efforts to strengthen surgical systems and improve access to safe surgical, obstetric, and anesthesia care.

## Methods

### Study design

This study is a convergent mixed-methods evaluation [11] of the SS2020 program in Cambodian intervention hospitals. This evaluation integrates quantitative data from a prospective, observational study and qualitative data gathered via semi-structured focus groups. This mixed-methods approach was adopted to provide a more comprehensive understanding of the depth and scale of impact of the intervention.

### Intervention

**Figure 1.**
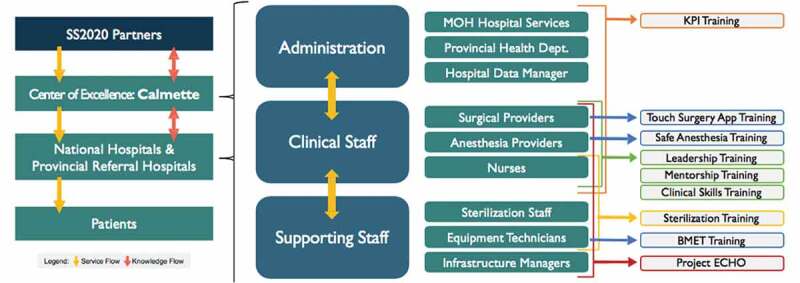
Safe Surgery 2020 flow of service and knowledge through Center of Excellence with link to trainings

In Cambodia, a hub-and-spoke model for implementation of SS2020 was adopted, wherein a national hospital was established as a Center of Excellence that could cascade trainings, build mentor-mentee relationships, and provide ongoing engagement with other hospitals across the country. Calmette Hospital, a premier national hospital in Phnom Penh, served as the Center of Excellence and hub. SS2020 was implemented over 12 months, starting in Calmette and expanding to five provincial and two national hospitals over two rounds of trainings in partnership with the MoH (Table 1). Calmette led trainings for three of these facilities, including both national hospitals. Trainings were implemented in 2019 and were followed by several months of virtual and in-person mentorship. Approximately 200 total surgical staff were trained and included surgeons, anesthetists, nurses, medical directors, and other surgical support staff, as well as representatives of the MoH. The goal was to empower leadership at the hub to support interventions at spoke hospitals through mentorship (Figure 1). Calmette was advised by SS2020 on curriculums and materials to reflect best practices and provide technical support to the provincial facilities.

**Table 1.** Facility characteristics^1^

Facility Type	Average # of monthly surgical procedures	# of surgical, anesthetic, and obstetric providers	# of facility staff attending SS2020 trainings^2^
NationalHospitals	1161	84	49
	993	85	28
	283	47	31
Provincial Hospitals	96	21	25
	302	25	19
	62	21	13
	159	32	18
	113	17	14

^1^Characteristics were self-reported by facilities at end of program implementation in late 2019/early 2020

^2^Some SS2020 trainees served in administrative or leadership positions and were not staffed on respective facilities’ surgical teams

SS2020 was delivered by a multi-stakeholder partnership that convened leaders from academic, private, and public sectors to increase access to safe surgery in Cambodia. Lead partners included Dalberg Advisors, Assist International, Harvard University’s Program in Global Surgery and Social Change (PGSSC), and Jhpiego, an affiliate of Johns Hopkins University. Each partner undertook a different component of the intervention, linking innovations with global expertise and local experience. Further support from Sterile Processing Education Charitable Trust (SPECT) and World Federation of Societies of Anesthesiologists (WFSA) was utilized to address specific needs for Cambodia. In-country partners included the MoH, Calmette Hospital, and the University of Health Sciences. These alliances were critical to establish a sustainable and scalable approach for the program.

The multicomponent intervention included equipment donations and nine partner-led trainings followed by mentorship implemented over a period of one year. These trainings were tailored to meet the specific needs of the Cambodian context and were approved by the MoH. A mentorship model was implemented, with Calmette co-facilitating many of the initial trainings and providing follow-up supportive mentorship with lead partners. Following is a description of each of the trainings:

1. *Key Performance Indicators (KPI) Data Training* was conducted by the PGSSC to understand the role of monitoring and evaluation within surgery, providing the mechanisms to collect, aggregate, and report high quality surgical data and KPIs.
2. *Leadership and Mentorship Training* was conducted by Jhpiego to provide team-based leadership skills for surgical team members to become agents of change at their facility and in their community by implementing quality improvement programs.
3. *Safe Cesarean Birth Clinical Skills/Patient Safety Training* was conducted by Jhpiego to provide interactive, evidence-based clinical skills for the practice of safe and infection free cesarean sections by the surgical teams.
4. *Touch Surgery Virtual Reality Clinical Skills Training* was conducted by Assist International to provide training on surgical skills utilizing the mobile phone application, Touch Surgery, which helps to prepare for surgery, learn new procedures, and test surgical knowledge.
5. *Safe Anesthesia Training* was conducted by WFSA to increase the capacity of all levels of anesthesia providers in the delivery of safe anesthesia for obstetric care.
6. *Surgical Equipment Sterilization Training* was conducted by SPECT to improve knowledge and practices of sterile processing of surgical equipment to reduce the risk of infection.
7. *Bio-medical Engineering Training* (BMET) was conducted by Assist International to provide training for technicians to operate, maintain, and repair medical equipment through education and both practical and professional experience.
8. *Project ECHO* was set up by Assist International and utilized in collaboration with Jhpiego, WFSA, SPECT and Calmette to be set up as a platform for multipoint video conferencing at all participating facilities to provide guided practice and remote mentorship.
9. *Equipment Donations and Clinical User Training* was conducted by Assist International to impart basic user training and clinical applications training for introduction to, and usage of, donated medical equipment including anesthesia machines, anesthesia monitors, pre and post-op patient monitors, and ventilators.

One of the key cross-cutting topics covered in almost all of these trainings was the World Health Organization’s *Surgical Safety Checklist (SSC)*, which has been widely used since a 2009 study provided evidence of its use leading to reduced mortality and morbidity among surgical patients [12]. Each component of the checklist and its ideal application in a surgical setting were taught to trainees.

### Data collection and analysis

#### Quantitative arm

Quantitative data for this mixed methods evaluation were collected prospectively via surgical observations conducted at baseline and endline, between February 2019 and January 2020, in 8 intervention hospitals. The objective of the surgical observations was to assess adherence to global standards of surgical care, primarily focusing on items found on the SSC. The items assessed included adherence to safety practices, teamwork and communication, and other SS2020 program-specific items.

The tool used for these observations was adapted by PGSSC from previously implemented tools [13,14] and was administered by local, trained medical students to assess completion of 20 surgical performance items. Fourteen of these items are commonly found on the SSC, while the remaining 6 were added as program-specific items. Power and sample size calculations performed using nQuery [15] estimated that a minimum total of 64 surgical cases at baseline and 64 cases at endline were necessary to provide over 80% power to detect a 5% improvement. To meet this sample size, data collectors observed as many surgeries as feasible in all intervention hospitals over approximately 10 days at both baseline and endline. During these procedures, collectors closely monitored the operating room and recorded all relevant observations on the 44-item observation tool. To ensure data quality, collectors received extensive training, participated in pilot collection prior to baseline, and were subjected to regular data quality checks from study staff.

Data were analyzed using Stata (version 15.0, StataCorp LLC. College Station, TX) [16]. First, the pre-post percent change for each of 20 indicators was calculated. Then, a generalized estimating equation (GEE) was conducted for each percentage change to calculate an odds ratio and a p-value. The GEE was intended to test the significance of the percentage change and predict the odds of an indicator’s outcome at population level. For further exploration of results, indicators were disaggregated by hospital type to assess for any trends in pre-post change between provincial and national hospitals.

#### Qualitative arm

Focus group discussions (FGDs) were conducted at endline in each of the 8 intervention hospitals. The objective of the FGDs was to understand the overall perceived impact of SS2020 programs and to identify the facilitators and barriers to positive change. Semi-structured group interviews were conducted in Khmer in each intervention hospital and were facilitated by two male Khmer-speaking researchers. Both researchers were professionals with academic training in qualitative data collection and several years of experience leading and interpreting interviews and focus groups locally; they were recruited by the study team using local professional networks. PGSSC staff, jointly with the researchers, recruited participants that attended at least one SS2020 training. A final convenience sample was selected based on availability of participants, with priority given to those who attended multiple trainings. Participants were informed that individual responses would remain anonymous and that researchers were external partners with unbiased research interests in the program and were committed to creating a safe environment for discussion. A total of 15 groups were conducted, 2 in each hospital except for the hub hospital, where only one large group discussion took place. All FGDs were recorded, transcribed, and translated from Khmer to English. Detailed field notes with observations and reflections were also recorded by PGSSC staff and researchers.

The grounded theory approach – a systematic and inductive method of analyzing qualitative data – was adopted to analyze the FGDs [17]. Two authors of this study (SB, JI) developed a codebook and independently coded the longest transcript in NVivo 12 [18]. The codebook was refined after iterative discussions between authors and a high inter-rater reliability (IRR) score between coders was calculated (kappa = 0.81) [19]. The authors proceeded to divide the remaining transcripts and code in NVivo with the final codebook and summarized key themes of the data. The codes were then reviewed for their key findings and were integrated into five broader themes: knowledge gain perceptions, individual behavior change experiences, institutional change and patient impact, perspectives on the hub-and-spoke model, and path to sustainability. These themes were partially informed by the Kirkpatrick framework for evaluation, a commonly used model to evaluate the impact of training programs [20], which the lead authors of this study recently applied to another training program in safe surgery in Ethiopia [21]. The 32-item consolidated checklist for reporting qualitative research (COREQ) was followed throughout analysis write-up.

#### Ethics approval

Ethics approvals were procured for this study from Harvard Medical School Institutional Review Board, and the Cambodian MoH National Ethics Committee for Health Research. For greater transparency and accountability, additional approvals from the directors of all intervention hospitals were also procured. Furthermore, prior to data collection, assent from all patients involved in surgical observations and all surgical staff involved in qualitative data collection was obtained.

## Results

### Adherence to surgical safety checklist items

Surgical observations were conducted in all eight intervention hospitals. Five intervention sites were regional referral hospitals and three were national hospitals. A total of 202 procedures were observed at baseline and 231 at endline. The most commonly observed surgeries at both baseline and endline were caesarian procedures, 47% and 44%, respectively, followed by appendectomies (10%, 16%) and fracture repairs (8%, 14%). Collectively known as the Bellwether Procedures, these are the most commonly performed major surgical operations in the intervention hospitals.

Table 2 shows changes in safety, teamwork, and communication practices before and after the SS2020 intervention. A total of 20 indicators were calculated with the surgical observations data. Of these, 19 show a positive change from baseline to endline; 18 of these changes were statistically significant. Among the highest performing indicators are safety items, including administration rate of prophylactic antibiotics (+27.5%, *p* < .001), rate of post-operative decontamination of all instruments (+23.5%, *p* < .001), and rate of vaginal cleansing with povidone iodine for cesarean sections (+21.8%, *p* < .001). Among the lowest performing indicators are communication items; data show a statistically insignificant increase of 1.3% (*p* = .184) in the rate of surgeon’s discussion on patient-specific concerns, and 3.2% (*p* = .005) increase in rate of discussion on patient post-op recovery.

**Table 2.** Completion rates (%) of performance indicators for surgical procedures observed at baseline and endline in 8 hospitals

Key Outcome Indicators	Baseline		Endline		Percent Change(Baseline-Endline)	Odds Ratio*	95 % CI	p-value
	*N = 202*		*N = 231*					
	n	%	n	%	%			
**Safety Items**								
1. Patient Identity, Consent, and Procedure Confirmation Rate	85	42.3	110	47.6	5.3	1.24	[1.11, 1.38]	< .001
2. Appropriate Prophylactic Antibiotic Administration Rate	124	61.7	206	89.2	27.5	5.12	[4.57, 5.73]	< .001
3. Pulse Oximetry Usage Rate	191	94.6	228	98.7	4.1	4.38	[3.41, 5.62]	< .001
4. Rate of Surgical Instruments Free of Visible Soil	120	59.4	172	74.5	15.1	1.99	[1.79, 2.22]	< .001
5. Rate of Observed Chemical Sterilization Tape	104	52	138	59.7	7.7	1.37	[1.23, 1.53]	< .001
6. Rate of Appropriate Operative Site Cleansing	192	95	229	99.1	4.1	5.96	[4.54, 7.83]	< .001
7. Rate of Vaginal Cleansing with Povidone-Iodine (for caesarean sections only)	20	21.3	25	43.1	21.8	2.80	[2.02, 3.90]	< .001
8. Instrument, Sponge, and Needle Count Verifications	98	48.5	155	67.7	19.2	2.22	[2.01, 2.46]	< .001
9. Rate of Post-op Decontamination of All (used and unused) Instruments	145	72.1	225	97.4	25.3	14.48	[12.58, 16.67]	< .001
**Communication Items**								
10. Rate of Discussion on Risk for Blood Loss	35	17.4	4	33	15.6	2.34	[2.04, 2.68]	< .001
11. Rate of Discussion on Risk for Airway Difficulty/Aspiration	36	18	58	25.1	7.1	1.53	[1.32, 1.76]	< .001
12. Rate of Discussion on Sterility of Instruments and Equipment	70	34.7	102	44.2	9.5	1.49	[1.34, 1.66]	< .001
13. Rate of Surgeon’s Discussion on Patient-specific Concerns	27	13.5	34	14.8	1.3	1.11	[0.95, 1.30]	0.184
14. Rate of Anesthetist’s Discussion on Patient-Specific Concerns	20	9.9	34	14.7	4.8	1.57	[1.32, 1.87]	< .001
15. Rate of Surgeon’s Discussion on Anticipated Procedure level of Difficulty and Duration	47	23.3	95	41.1	17.8	2.30	[2.04, 2.60]	< .001
16. Rate of Discussion on Equipment/Instrument Problems During Surgery	11	5.5	41	17.7	12.2	3.71	[2.94, 4.68]	< .001
17. Rate of Discussion on Patient’s Post-op Recovery	45	22.3	59	25.5	3.2	1.20	[1.06, 1.36]	0.005
**Checklist Utilization Indicators**								
18a. Rate of Surgical Safety Checklist Utilization (Any Method)	140	69.3	196	84.8	15.5	2.48	[2.21, 2.78]	< .001
18b. Rate of Surgical Safety Checklist Utilization (On Paper, Read Aloud)	45	32.1	56	28.6	−3.5	0.84	[0.69, 1.03]	0.089
18c. Rate of Adherence to Surgical Safety Checklist Items	1	0.5	9	3.9	3.4	8.15	[3.95, 16.82]	< .001

*Generalized Estimating Equations (GEE) estimates population average effects; it provides estimates that predict the odds of an outcome on a population level. Example statement: The odds of prophylactic antibiotic being administered before incision are increased five-fold after exposure to SS2020 interventions; the change is statistically significant (*p* < .001).

For overall SSC utilization indicators, data were analyzed using three different methods: (1) rate of checklist utilization including any attempt made by surgical staff to use it during sign in, time out, or sign out; (2) rate of checklist utilization including only attempts where surgical staff adequately used it as instructed, on paper, and read aloud at appropriate intervals; and (3) rate of adherence to key checklist items. Increases were seen in all three indicators, with any attempt at checklist utilization seeing the largest increase (15.5%, *p* < .001).

When disaggregated by hospital type (national vs. provincial), the data show greater improvement (greater percentage change) in provincial hospital for 15 of 20 indicators (Table 3). For the higher performing indicators, most of the positive change is concentrated in provincial hospitals, though baseline numbers are generally comparable between the two groups. Prophylactic antibiotic administration rate increased by 35.8% in provincial hospitals, and only 13.3% in national hospitals. For rate of vaginal cleansing, positive change is seen only in the provincial hospitals (18.8%). Any attempts made to use the SSC was also higher in provincial hospitals (25.5% v. 7.7%). Additionally, national hospitals saw decreases in performance for a few communication indicators, including rate of discussion on patient-specific concerns.

**Table 3.** Percent change of performance indicators for surgical procedures observed at baseline and endline, by hospital type

	National Hospitals*			Provincial Hospitals		
	% Baseline	% Endline	% Change	% Baseline	% Endline	% Change
**Safety Items**						
1. Patient Identity, Consent, and Procedure Confirmation Rate	23.6	16.7	−6.9	44.4	60.6	16.2
2. Appropriate Prophylactic Antibiotic Administration Rate	65.3	78.6	13.3	59.6	95.4	35.8
3. Pulse Oximetry Usage Rate	97.2	100	2.8	90.9	97.2	6.3
4. Rate of Surgical Instruments Free of Visible Soil	48.6	71.4	22.8	54.5	79.8	25.3
5. Rate of Observed Chemical Sterilization Tape	55.6	73.8	18.2	34.7	39.4	4.7
6. Rate of Appropriate Operative Site Cleansing	97.2	100	2.8	91.9	98.2	6.3
7. Rate of Vaginal Preparation with Providone-Iodine (for caesarean sections only)	23.1	14.7	−8.4	27.5	46.3	18.8
8. Instrument, Sponge, and Needle Count Verifications	19.4	50.6	31.2	61.6	88.1	26.5
9. Rate of Post-op Decontamination of All (used and unused) Instruments	27.8	98.8	71	95.9	95.4	−0.5
**Communication Items**						
10. Rate of Discussion on Risk for Blood Loss	9.7	14.3	4.6	16.3	46.3	30
11. Rate of Discussion on Risk for Airway Difficulty/Aspiration	2.8	1.2	−1.6	14.4	47.7	33.3
12. Rate of Discussion on Sterility of Instruments and Equipment	4.2	19	14.8	42.4	55	12.6
13. Rate of Surgeon’s Discussion on Patient-specific Concerns	19.4	7.2	−12.2	7.2	19.3	12.1
14. Rate of Anesthetist’s Discussion on Patient-Specific Concerns	15.3	1.2	−14.1	6.1	26.6	20.5
15. Rate of Surgeon’s Discussion on Anticipated Procedure level of Difficulty and Duration	4.2	11.9	7.7	28.3	60.6	32.3
16. Rate of Discussion on Equipment/Instrument Problems During Surgery	0	11.9	11.9	9.3	27.5	18.2
17. Rate of Discussion on Patient’s Post-op Recovery	15.3	15.5	0.2	25.3	33.9	8.6
**Checklist Utilization Indicators**						
18a. Rate of Surgical Safety Checklist Utilization (Any)	54.2	61.9	7.7	72.7	98.2	25.5
18b. Rate of Surgical Safety Checklist Utilization (On Paper, Read Aloud)	0	9.6	9.6	25	26.2	1.2
18c. Rate of Adherence to Surgical Safety Checklist Items	0	0	0	1	8.3	7.3

*National Hospitals category excludes cases from Hub Hospital

### Facilitators and barriers to implementation

During the endline, 15 FGDs were conducted across all 8 intervention hospitals, with a total of 80 participants. Each FGD had a minimum of three participants, and a maximum of nine. Of these, 77.5% and 22.5% were male and female, respectively. The average number of years participants have worked in their respective hospitals is 12, and their average age is 38. A majority of participants were surgeons, anesthetists/anesthesiologists, or other medical doctors (55%). A large proportion were nurses (25%) or technical support staff (18%). The FGD data is best summarized into five key themes: knowledge gain perceptions, individual behavior change experiences, institutional change and patient impact, perspectives on the hub-and-spoke model, and path to sustainability. Table 4 provides key findings and illustrative quotations that emerged from the FGDs. Quotations have been edited for conciseness.

**Table 4.** Qualitative results: key findings and illustrative quotations

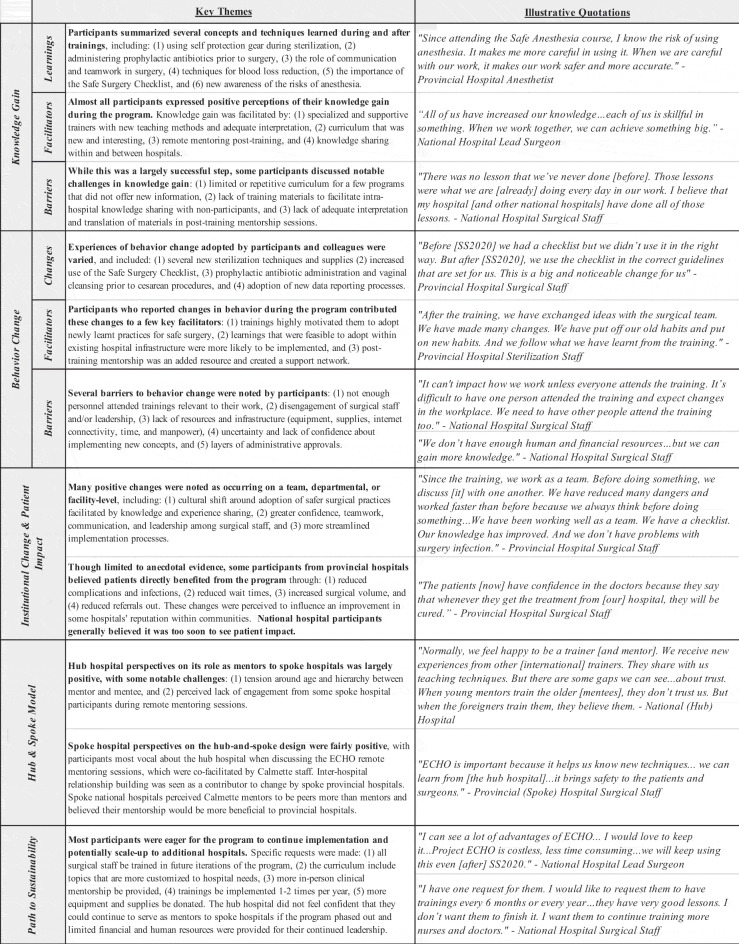

*Knowledge Gain*: Almost all participants expressed positive perceptions of their knowledge gain during the program. Specialized and supportive trainers, ECHO remote mentoring post-training, and knowledge sharing facilitated knowledge gain for key concepts such as instrument sterilization, antibiotic administration, and teamwork in surgery. A few barriers to this domain also emerged, including limitations of translation in post-training mentorship sessions.

*Individual Behavior Change*: Experiences of behavior change adopted by participants and colleagues varied by training type, with respondents reporting improvements in practice for equipment sterilization, antibiotic administration, use of the SSC, and other areas of clinical practice. Participants who reported changes in behavior during the program attributed these changes to a few key factors, including the ongoing mentorship after initial trainings. Lack of resources and infrastructure and administrative delays were identified as some of several barriers to behavior change.

*Institutional Change and Patient Impact*: Many positive changes were noted as occurring on a team, departmental, or facility-level, including a cultural shift around adoption of safer surgical practices. This was facilitated by knowledge and experience sharing, as well as greater confidence, teamwork, communication, and leadership among surgical staff. Respondents from national hospitals stressed that it was too soon to see any direct patient impact due to the program. Regional hospitals, however, provided some anecdotal evidence of what they perceived to be patient impact, such as increased surgical volume due to the local community’s growing trust in their facilities.

*Hub and Spoke Mentorship Model*: Respondents from the spoke hospitals had favorable views about the hub-and-spoke model and their respective mentor/mentee relationships. Spoke regional hospitals were most vocal about the hub hospital (Calmette) when discussing the ECHO remote mentoring sessions, while spoke national hospitals stressed that they perceived Calmette mentors to be peers and believed that their mentorship is more beneficial to regional hospitals.

*Path to Sustainability*: Most participants were eager for the program to continue implementation and potentially scale-up to additional hospitals. Several specific requests for improvements were made, and included customized curriculums per hospital needs, increased frequency of trainings, and more equipment donations. The hub hospital respondents noted interest in continuation of their role as mentors pending adequate allocation of financial and human resources.

While several challenges were identified during the FGDs, respondents had generally positive perceptions of the SS2020 program and were eager to not only continue it but to also expand its scope and reach.

## Discussion

This paper describes the mixed-methods assessment of an innovative safe surgery training and mentorship intervention in Cambodia. Results show that SSC use increased and that the biggest improvements in adherence were in safety items on the checklist more so than teamwork and communication items. The qualitative component of the study explored themes in five key domains. The knowledge gain step was largely successful; individual behavior change step faced several challenges; institutional change and patient impact steps saw the early stages of progress. The hub and spoke mentorship model of the program was perceived to be favorable for provincial hospitals and all hospitals expressed interest in sustaining and scaling up the program.

### Interpretation of results

The FGD results offer contextualization of several key results of the quantitative component of the assessment. During the FGDs, certain items from the SSC were repeatedly highlighted by respondents. The administration of prophylactic antibiotics, vaginal cleansing with povidone iodine, and equipment sterilization were key SSC items frequently discussed by surgical staff and were also the quantitative indicators that saw the largest improvements between baseline and endline.

When disaggregated by hospital type, results showed greater progress in provincial hospitals than in national hospitals. FGD results offer a few potential reasons for this discrepancy. Spoke national hospitals believed the program, especially the mentorship component, was likely more beneficial to provincial hospitals; they did not believe they had as much to learn from hub mentors. This attitude may have potentially contributed to some level of resistance to learning and adopting change. Additionally, less progress in national hospitals may have been caused by the dilution of training benefits due to larger team sizes; one of the challenges noted in the discussions was that not all surgical staff were invited to participate in the trainings. This discrepancy in experiences between facility types highlights the need for different approaches that are customized for hospital needs.

Focus group discussions also highlighted the diverse impact of various trainings. The discussions around some of the more highly technical and less attended trainings, such as BMET and Clinical User trainings, were often less insightful than the trainings with greater number of participants. Most programs were discussed positively during the FGDs; some, however, had less favorable opinions. The introduction of the *Touch Surgery* application was not seen as particularly useful by respondents and most respondents did not report using it after the initial training. However, programs such as SPECT training, the leadership and mentorship training, as well as the clinical skills trainings were seen as informative, relevant, and important. Selective implementation of these programs should be considered in scaling of SS2020.

### Interpretation of results in the context of literature

Recent evaluation studies of SS2020 implementation in Tanzania have yielded similar interpretations of program impact. An earlier iteration of the hub-and-spoke mentorship model was first implemented in Tanzania in 2018 and also saw improved self-reported SSC utilization rates in the program’s first year [22]. Another study indicated that higher performing facilities had a more holistic approach to change that included prioritization of safety practices, teamwork and communication; lower performing facilities tended to delay prioritization of communication [23]. Our results in Cambodia showed higher levels of improvement among safety practices and less in teamwork and communication, which reflect the challenges of the short program timeline.

Other safer surgery evaluations note similar findings in high-income countries. In 2016, Singer et al. conducted an SSC utilization study in 10 American facilities that found greater adherence to safety items (or ‘procedural checks’) than teamwork and communication items (or ‘conversation prompts’). Authors concluded that this discrepancy was directly related to the importance of team and surgeon buy-in and consistent communication [24].

The results of other training programs in low-and-middle income countries that develop surgery and anesthesia workforce reflect some of the findings of this study. A laparoscopic surgery training implemented in Mongolia led to improved confidence of participants and adoption of self-sustaining practices, as well as increased trust of the community [25]. Newton et al. evaluated the implementation of parallel anesthesia and surgical provider trainings in East Africa which resulted in the increase of surgical cases fourfold [26]. Additional studies conducted in East and Central Africa on the impact of the Safer Anaesthesia from Education (SAFE) program also found improved knowledge, change in practice, and improved communication [27,28]. The studies discussed above show varying levels of success on short (<1 year) and long (3–5 years) training timelines. Shorter programs saw improvement in knowledge gain, while longer programs saw additional improvements in clinical outcomes.

### Implications and recommendations

The results of this study provide evidence that change in surgical ecosystems can be achieved on a short timeline with limited resources. The hub and spoke mentorship model can be successful in improving knowledge and changing behavior around safe surgery. Workforce development is important to improving surgical systems, but greater financial and human resources are needed. The MoH’s support in adopting, leading, and scaling of trainings is crucial to the continued success of safe surgery interventions in Cambodia.

Safe surgery implementing partners and the MoH can apply the lessons learned in this evaluation to design future hub and spoke training and mentorship models in surgery and anesthesia workforce development. Allotting adequate timelines to meet long-term objectives is crucial. Short-term programs such as the one discussed in this paper may see changes in knowledge gain and behavior change in less than one year, but a multi-year commitment will likely see additional layers of institutional change and patient impact. Collaboration with a hub hospital that has the motivation and resources to serve as a leading institution will be crucial to any hub and spoke training and mentorship program. Selection of spoke facilities from the hub’s existing referral networks is also recommended and will build stronger inter-hospital relationships.

Furthermore, setting a clear sustainability plan that is agreed to by all implementers, including the hub hospital, at the onset of any program is imperative. Facilitation of ongoing post-training mentorship with a balanced integration of virtual and in-person sessions should be considered. The complex skills required for surgery and anesthesia require a blended model to better enable learning. Some of these skills, such as knowledge sharing and decision-making, are well suited to virtual learning, while other skills, such as organization of clinical services and teamwork building, are better supported through in-person learning.

Greater customization of the training and mentorship curriculum should be considered where feasible, to fit the local context, especially discerning between the needs of national and regional hospitals. Removal of unfavorable components of the intervention, such as the use of the *Touch Surgery* application, is recommended. Furthermore, training all surgery and anesthesia providers instead of a subset is preferable wherever resources allow it. Implementers should address infrastructure gaps in parallel to workforce trainings. Additional supply and equipment distributions and trainings, as well as improved internet connectivity options would have greatly benefitted SS2020 Cambodia. Finally, since these results are not necessarily generalizable to facilities in rural settings, further discussion with the MoH on how to successfully customize the curriculum in the rural context is needed.

### Limitations

This study has limitations. A convenience sample was used for the quantitative surgical observations data collection and due to small sample size, individual facility-level analysis pre and post intervention was not feasible. Additionally, while social divergence within surgical teams may have contributed to less favorable results around teamwork and communication, this was not fully investigated in this study due to lack of resources and concerns with anonymity. The inclusion criteria for the FGDs excluded different staff perspectives, including those that may have been involved in mentorship activities but were not directly trained at the onset of the program. Additionally, FGD results were subject to recall bias and social desirability bias among participants.

## Conclusions

The results of this evaluation show notable progress in safe surgery knowledge gain and behavior change among surgical teams in SS2020 intervention hospitals in Cambodia. While these improvements were seen on a short timeline, institutionalized change with evident positive patient impact requires additional time and resources. The MOH should be encouraged to heed lessons learned from this program and adopt the curriculum for regional and national scaling. Building on the gains addressed in this paper will be crucial for long-term sustainability of the safer surgery movement in Cambodia.

## Paper context

The availability of safe surgical care is an ongoing challenge in low-resource settings. An innovative safe surgery workforce training and mentorship intervention was implemented in eight hospitals in Cambodia. A mixed-methods evaluation of this intervention was implemented. Results provide evidence that this model of building knowledge and capacity among the surgical workforce should be adapted and implemented in similar contexts.
